# An alarming rise of non-*albicans Candida* species and uncommon yeasts in the clinical samples; a combination of various molecular techniques for identification of etiologic agents

**DOI:** 10.1186/s13104-019-4811-1

**Published:** 2019-11-29

**Authors:** Monireh Taei, Mostafa Chadeganipour, Rasoul Mohammadi

**Affiliations:** 1 Department of Medical Parasitology and Mycology, School of Medicine, Isfahan University of Medical Sciences, Isfahan, Iran; 2 Infectious Diseases and Tropical Medicine Research Center, Isfahan University of Medical Sciences, Isfahan, Iran

**Keywords:** Non-*albicans Candida* species, Uncommon yeasts, *Trichosporon asahii*, *Pichia terricola*, Identification, Molecular techniques

## Abstract

**Objective:**

Yeasts are unicellular microorganisms may cause systemic infection in immunocompromised patients. The aim of this study was to identify yeast strains isolated from clinical specimens using molecular techniques.

**Results:**

A total of 202 yeast strains isolated from 341 clinical samples between February 2017 and May 2019. All clinical isolates were identified using phenotypic and molecular tests including PCR–RFLP, duplex-PCR, multiplex-PCR, and PCR-sequencing. The most yeast fungal isolates were obtained from urine (66.8%), nail (9.4%), skin lesion (7.9%), bronchoalveolar lavage (5.9%), and blood (3.9%). One hundred and twenty-one *Candida* species were identified as non-*albicans* versus 76 *Candida albicans. Trichosporon asahii,* and *Pichia terricola* were uncommon non-*Candida* yeasts isolated from urine samples. For the first time, we isolated *P. terricola* as etiological agent of urinary tract infection in a pregnant female. Since *Candida* species show different levels of resistance to antifungal agents, precise identification of clinical isolates is critical for better treatment of infection.

## Introduction

*Candida* species are the most common cause of fungal infections worldwide. They are the third most dominant cause of healthcare-related infections [1]. *Candida albicans* is the most common species in humans; however, the increasing of non-*albicans Candida* (NAC) species have been recognized significantly during the last two decades [2–4]. The most NAC infections are caused by *C. glabrata*, *C. tropicalis, C. parapsilosis, C. dubliniensis, C. guilliermondii, C. krusei*, and *C. kefyr* [5–7]. The growing number of NAC species might be connected to former exposure to polyenes and azoles, use of indwelling catheters, malignancies, age, the improved biochemical and molecular diagnostic methods in laboratories, and geographical regions [8, 9]. As compared with *C. albicans*, this epidemiological pattern towards NAC species with high MICs of azoles and triazoles, appears among high-risk patients due to the common and prolonged use of antifungal agents [10]. High mortality rate (30-50%), along with raising resistance to antifungal agents among NAC species, imposes serious medical and economic problems to the society [7, 11]. The raising frequency of NAC species has been repeatedly reported from different areas worldwide, for example, *C. tropicalis* is frequently isolated in Asia and South America and *C. glabrata* has a high frequency in North and Central Europe and United States of America specially, among the elderly people [12]. Due to the increase reports of uncommon yeast infection and their antifungal resistance by physicians in our region, the present study was undertaken to investigate the frequency of various *Candida* species and rare yeasts collected from fungal infections in Kashani university hospital and Shefa Lab (referral medical mycology laboratory), in Isfahan, Iran, by combination of miscellaneous molecular techniques.

## Main text

### Materials and methods

#### Patients and sampling

A total of 341 suspected cases (75 male and 266 female) referred to the Kashani university hospital and a referral medical mycology laboratory (Shefa Lab.), in Isfahan, Iran, from February 2017 to May 2019. Demographic data were documented for each subject. Excluding criteria were considered for the patients who had taken antifungal agents during the last week.

#### Primary screening

All specimens were examined by direct microscopy with 10% Potassium Hydroxide (KOH 10%), sub-culture on sabouraud dextrose agar (Biolife, Italy), and CHROMagar *Candida* (France).

### Molecular methods as secondary screening for identification of *Candida* species

#### Polymerase chain reaction-restriction fragment length polymorphism (PCR–RFLP) for identification of six prevalent species of *Candida*

This screening was done in an iterative manner (Additional file 1: Figures S1–S6).

Genomic DNA of isolates was extracted and ITS1-5.8SrDNA-ITS2 region was amplified. Briefly, the ITS1-5.8SrDNA-ITS2 region was amplified by a PCR mixture including 5 μL of 10× reaction buffer, 0.4 mM dNTPs, 1.5 mM MgCl_2_, 2.5 U of Taq polymerase, 30 pmol of both ITS1 (5′-TCC GTA GGT GAA CCT GCG G-3′) and ITS4 (5′-TCC TCC GCT TAT TGA TAT GC-3′) primers and 2 μL DNA in a final volume of 50 μL. The PCR cycling conditions were: an initial denaturation phase at 94 °C for 5 min, followed by 32 cycles of denaturation at 94 °C for 30 s, annealing at 55 °C for 45 s, and extension at 72 °C for 1 min, with a final extension phase at 72 °C for 7 min. PCR products were digested with the restriction enzyme MspI (Fermentas, Vilnius, Lithuania). Five microliter of each PCR amplicons and 10 μL of RFLP products were separated by gel electrophoresis on 1.5% and 2% agarose gel (containing 0.5 μg/mL ethidium bromide), respectively. In this stage, all clinical isolates first grouped into 6 prevalent species as *Candida albicans, C. tropicalis, C. parapsilosis* complex, *C. glabrata, C. krusei*, and *C. guilliermondii* [13].

#### Duplex-PCR assay for distinction of *C. albicans* and *C. dubliniensis*

Species-specificity primers were used to identify *C. albicans* and *C. dubliniensis*. The sequences of these primers are as follows:

CALF (5′-TGGTAAGGCGGGATCGCTT-3′) and CALR (5′-GGTCAAAGTTTGAAGATATAC) for *C. albicans*, and CDUF (5′-AAACTTGTCACGAGATTATTTTT) and CDUR (5′-AAAGTTTGAAGAATAAAATGGC-3′) for *C. dubliniensis*. The size of PCR products for *C. albicans* and *C. dubliniensis* are 100 bp, and 325 bp, respectively [14].

#### Multiplex-PCR for differentiation of *Candida glabrata* and its phylogenetically related species *Candida bracarensis* and *Candida nivariensis*

Targeting the ITS1 region, reverse primer UNI-5.8S (5′ ACCAGAGGGCGCAATGTG 3′) and forward primers GLA-f (5′ CGGTTGGTGGGTGTTCTGC 3′), NIV-f (5′ AGGGAGGAGTTTGTATCTTTCAAC 3′), and BRA-f (5′ GGGACGGTAAGTCTCCCG 3′), were applied for identification of *C. glabrata, C. nivariensis*, and *C. bracarensis*, respectively. Expected amplicon size for *C. glabrata, C. nivariensis*, and *C. bracarensis* are 379 bp, 293 bp, and 223 bp, respectively [15].

#### Multiplex-PCR for differentiation of *Candida parapsilosis* complex

Three pairs of primers including CPAF (5′ TTTGCTTTGGTAGGCCTTCTA 3′) and CPAR (5′ GAGGTCGAATTTGGAAGAAGT 3′), CORF (5′ TTTGGTGGCCCACGGCCT 3′) and CORR (5′ TGAGGTCGAATTTGGAAGAATT 3′), and CMEF (5′ TTTGGTGGGCCCACGGCT 3′) and CMER (5′ GAGGTCGAATTTGGAAGAATGT 3′), were used for identification of *Candida parapsilosis, C. orthopsilosis,* and *C. methapsilosis*, respectively. The size of amplicons for *Candida parapsilosis, C. orthopsilosis,* and *C. methapsilosis* are 379 bp, 367 bp, and 374 bp, respectively [16].

#### PCR-sequencing

All PCR products with no cutting site for the restriction enzyme *Msp*I considered as non-*Candida* yeasts, and were applied for sequence analysis. The non-*Candida* yeasts amplicons were purified using the ethanol purification method, and cycle sequencing reactions in forward direction were performed (Bioneer, South Korea). The sequencing products were analyzed with Chromas 2.3 (http://chromas.software.informer.com/2.4/), and were evaluated by using of NCBI BLAST searches against fungal sequences existing in DNA databases (http://blast.ncbi.nlm.nih.gov/Blast.cgi).

#### Data analysis

The results of phenotypic and genotypic tests were analyzed by Chi square and Fisher’s exact test in the SPSS version 23.

### Results

**Fig. 1:**
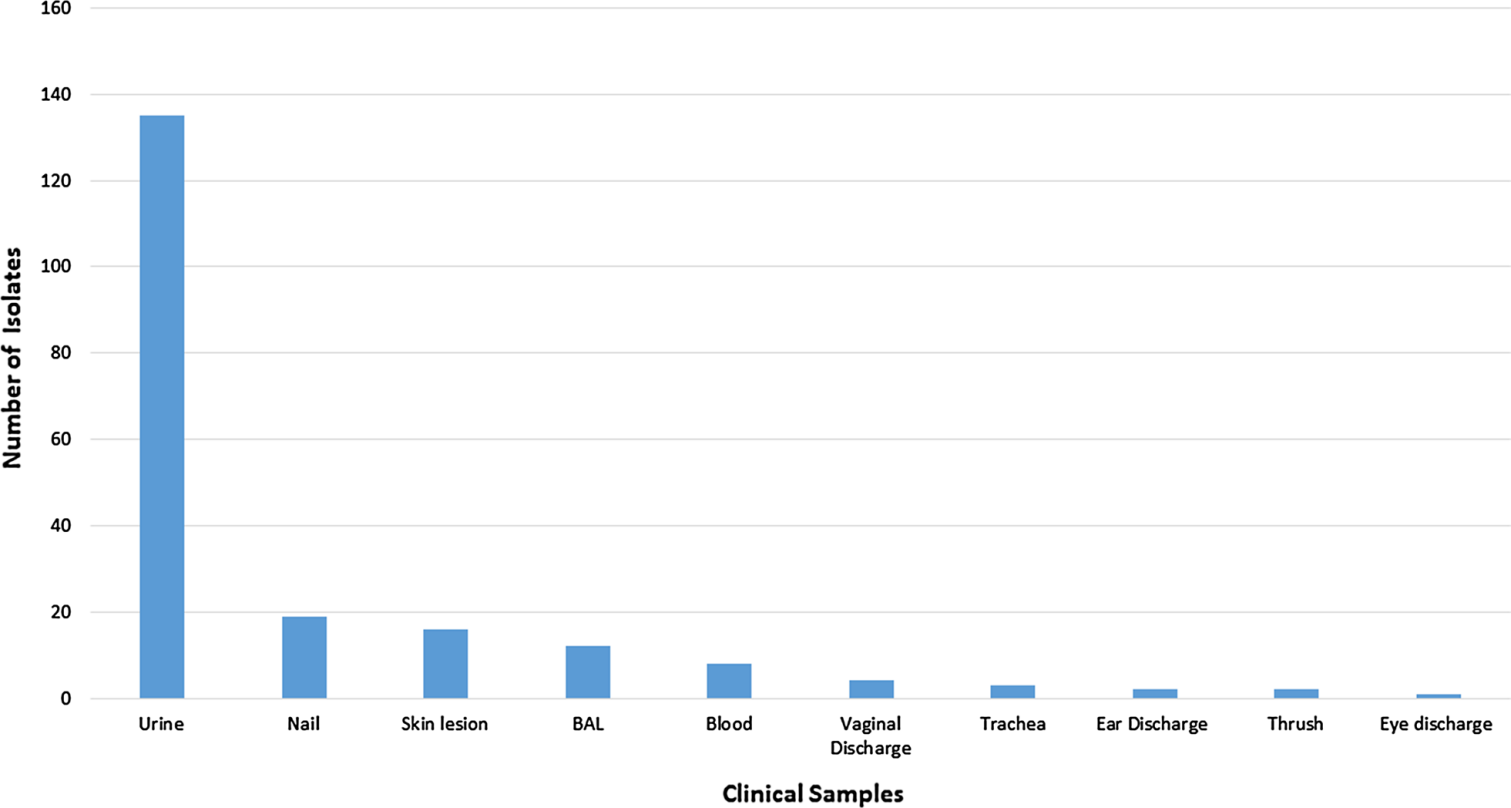
Various clinical samples infected to yeast species in the present study

**Fig. 2:**
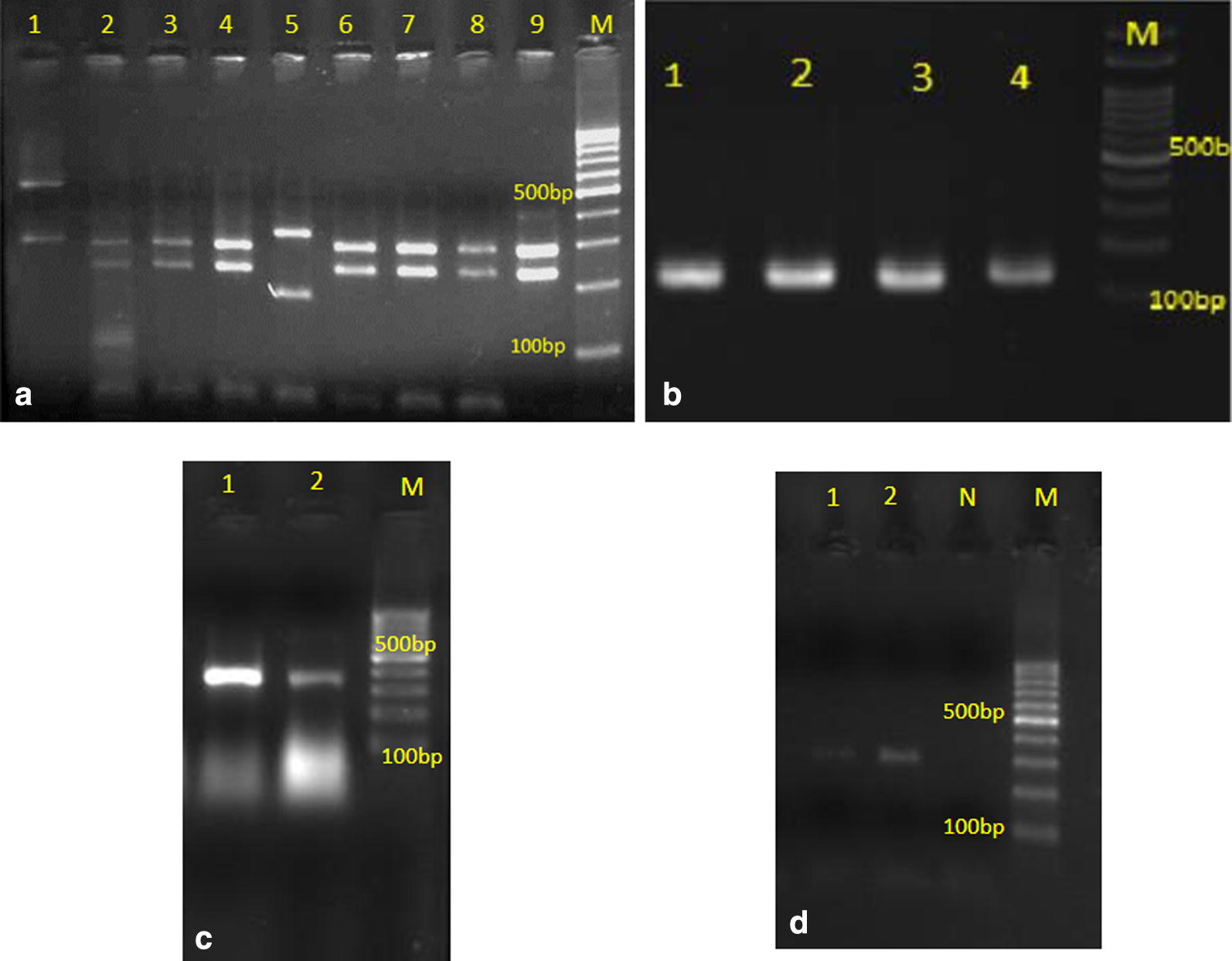
**a** Agarose gel electrophoresis of PCR–RFLP products of *Candida* isolates: lane 1 is *C. glabrata*, lanes 2–4, and 6–9 are *C. albicans*, and lane 5 is *C. tropicalis*; **b** Agarose gel electrophoresis of duplex-PCR for distinction of *C. albicans* and *C. dubliniensis*; lanes 1–4 are *C. albicans*; **c** multiplex-PCR for differentiation of *C. parapsilosis* complex; lanes 1, 2 are *C. parapsilosis*; **d** multiplex-PCR for differentiation of *C. glabrata* complex; lanes 1, 2 are *C. glabrata*; N is negative control; and M is 100 bp DNA size marker

Two hundred and two patients out of 341 suspected cases had yeast fungal infections (59.2%). Male to female sex ratio was 74/128. Age range of patients was 1–87 years with the median age range of 42.3. Diabetes (18.8%), pregnancy (18.3%), kidney stones (2.9%), burn (2.9%), and neoplasm (2.4%) were the most leading predisposing factors among patients. The most yeast fungal isolates were obtained from urine (66.8%), nail (9.4%), skin lesion (7.9%), bronchoalveolar lavage (5.9%), and blood (3.9%) (Fig. 1). The majority of patients hospitalized in ICU (9.9%), surgical ward (9.9%), neurological intensive care unit (NICU) (8.9%), infectious diseases ward (8.9%), and gynaecology ward (7.4%). Ninety-six cases (46.5%) were outpatients. Interestingly, 121 *Candida* species were identified as non-*albicans* versus 76 *Candida albicans* by molecular techniques (Fig. 2). Among NAC species, 92 *Candida glabrata* (76%), 8 *Pichia kudriavzevii* (teleomorph of the *Candida krusei*) (6.6%), 7 *C. kefyr* (5.7%), 6 *C. parapsilosis* (4.9%), 3 *C. tropicalis* (2.4%), 1 *C. dubliniensis* (0.8%), 1 *C. guilliermondii* (0.8%), 1 *C. famata* (*Debaryomyces hansenii*) (0.8%), 1 *Kluyveromyces marxianus* (teleomorph of the *Candida parapsilosis*) (0.8%), and 1 *C. orthopsilosis* (0.8%) were isolated from clinical samples. Fourteen isolates had no cutting site for the restriction enzyme *Msp*I and their amplicon sizes were under 500 bp. These clinical isolates were applied for cycle sequencing reactions in forward direction. Table 1 shows the frequency of *Candida* species in the present study among various clinical specimens in different wards of the hospital.

**Table 1:** The numbers of yeast isolates among various clinical samples

Yeast species	Clinical sample (number)	Ward (number)	Predisposing factor (number)	Molecular method for identification	Male to female sex ratio	Total number	Median age
*C. albicans*	Urine (39), Fingernail (11), Skin lesion (9), BAL (8), Vaginal discharge (3), Blood (3), Ear discharge (1), Eye discharge (1), trachea (1)	Outpatient (39), Gynaecology (10), ICU (9), Infectious Diseases (8), Surgical (3), Men (3), Neonatal (2), Dialysis (1), NICU (1)	Pregnancy (15), Diabetes Mellitus (10), Burn (2), Liver Cancer (1), Hepatitis (1), Leukemia (1), Asthma (1), Kidney stone (1), Lupus (1)	PCR–RFLP, Duplex-PCR	33/43	76	42.3
*C. glabrata*	Urine (72), Blood (5), Toenail (3), Skin lesion (5), trachea (2), Ear Discharge (1), Thrush (1), BAL (1), Vaginal discharge (1), Fingernail (1)	Outpatient (41), Surgical (13), NICU (12), ICU (7), Infectious Diseases (6), Gynaecology (5), CCU (3), General Neurology (3), Dialysis (1), Burn (1)	Diabetes Mellitus (20), Pregnancy (18), Kidney Stone (4), Burn (3), Lymphoma (1), Breast Cancer (1), Renal Failure (1), Splenectomy (1), Asthma (1), Tuberculosis (1), Constipation (1)	PCR–RFLP, Multiplex-PCR	29/63	92	43.3
*Pichia kudriavzevii (*teleomorph of the *Candida krusei)*	Urine (7), BAL (1)	Outpatient (3), Surgical (2), ICU (1), NICU (1), Neonatal (1)	Pregnancy (3), Kidney Stone (1), Tuberculosis (1)	PCR-sequencing	3/5	8	50.3
*C. kefyr*	Urine (6), Skin lesion (1)	Outpatient (4), Surgical (1), ICU (1), NICU (1)	Diabetes Mellitus (3), Burn (1)	PCR–RFLP	4/3	7	40.1
*C. parapsilosis*	Fingernail (2), Toenail (1), Skin lesion (1), Urine (1), Thrush (1)	Outpatient (4), NICU (2)	Diabetes Mellitus (1)	PCR–RFLP, Multiplex-PCR	2/4	6	40
*C. tropicalis*	Urine (3)	Outpatient (2), General Neurology (1)	Diabetes Mellitus (1), Leukemia (1)	PCR–RFLP	2/1	3	37.3
*C. dubliniensis*	BAL (1)	Infectious Diseases (1)	–	PCR–RFLP, Duplex-PCR	0/1	1	35
*C. guilliermondii*	Urine (1)	Outpatient (1)	–	PCR–RFLP	0/1	1	33
*C. orthopsilosis*	Fingernail (1)	Outpatient (1)	–	PCR–RFLP, Multiplex-PCR	0/1	1	41
*C. famata* (*Debaryomyces hansenii*)	Urine (1)	NICU (1)	–	PCR–RFLP	0/1	1	47
*Kluyveromyces marxianus* (teleomorph of the *Candida parapsilosis*)	BAL (1)	Infectious Diseases (1)	–	PCR-sequencing	0/1	1	65
*Trichosporon asahii*	Urine (4)	ICU (1), Dialysis (1), Surgical (1), Outpatient (1)	Diabetes Mellitus (3), Renal Failure (1)	PCR-sequencing	1/3	4	41.5
*Pichia terricola*	Urine (1)	Maternity (1)	Pregnancy (1)	PCR-sequencing	0/1	1	35

*ICU* intensive care unit, *NICU* newborn intensive care unit, *BAL* bronchoalveolar lavage

Fisher’s exact test revealed that the associations between the kind of clinical samples and yeasts were not statistically significant (*p *= 0.85).

### Discussion

Although *C. albicans* is the principal causative agent of candidiasis, the raising of NAC species resistant to antifungal drugs such as echinocandins and fluconazole in patients with nosocomial infections is concerning. Resistance to azoles is infrequent in *C. albicans* (< 5%), however, it is more current in *C. parapsilosis* (4–10%), *C. tropicalis* (4–9%), and *C. glabrata* (4–16%) [17]. Increasing of NAC species is due to the improved laboratory diagnosis, previous exposure to polyene and azole agents, use of indwelling medical devices, malignancies, growing number of immunocompromised patients, and long-term immunosuppressive and cytotoxic therapy in organ transplant recipients and cancer patients [9, 18]. It is usually believed that increasing use of azole treatments in clinics may be associated with the rise of NAC species [19]. The most patients infected to NAC, received empirical antifungals, whereas more *C. albicans* infections were treated when the diagnosis was confirmed. These findings may suggest that NAC infections are in worse clinical conditions [20]. In the last two decades, *C. albicans* was responsible for over 80% of etiological agents of all types of candidiasis [21], however, in the present study *C. albicans* isolated from almost 38.5% of *Candida* infections. *Candida glabrata* is obtained from 15 to 20% of *Candida* infections [22, 23], but we isolated 92 *C. glabrata* strains (46.7%) in the present investigation. In consistent with Singh et al. [24] we revealed a high prevalence of NAC species (61.4%), but *C. albicans* was the most repeatedly isolate (38.5%). The predominance of *C. glabrata* among NAC in the present investigation, is contrary to the prospective report by Chakrabarti et al. [25] where *C. tropicalis* was the most prevalent NAC species (42.1%). Furthermore, Bhattacharjee reported *C. tropicalis* as the most common NAC in a tertiary care hospital in Kolkata, India [26]. Kaur et al. and Al-Attas and Amro, reported a lower rate of *C. glabrata,* as well. They showed frequencies of (10%) and (11.1%) for *C. glabrata*, respectively [27, 28]. Interestingly, Pakshir et al. [29] showed *C. parapsilosis* as the most prevalent *Candida* species collected from fingernail infections (45.3%), even higher than *C. albicans* (23.7%). Non-*albicans Candida* infections may have more incidence among patients with allogeneic hepatocyte transplantation, neutropenia, hematologic malignancies, and severe immune suppression [30, 31] however, in our study, there was no difference between neutropenia and malignancies with *C. albicans* or NAC infections. Montagna et al. [32] found that *C. albicans* was more prevalent than NAC species in surgical wards, nevertheless, it was mainly isolated from gynaecology (n = 10), ICU (n = 9), and infectious diseases (n = 8) wards in the present study. According to PCR–RFLP using the restriction enzyme MspI, the differentiation between *C. albicans* and *C. dubliniensis* in *C. albicans* complex*, C. parapsilosis, C. orthopsilosis, and C. methapsilosis* in *C. parapsilosis* complex, and *C. glabrata, C. bracarensis* and *C. nivariensis* in *C. glabrata* complex is not possible, so we used effective molecular tests (duplex and multiplex-PCR) for distinguishing them. All strains in *C. glabrata* complex were *C. glabrata,* however, one *C. dubliniensis* and one *C. orthopsilosis* were isolated from *C. albicans* complex and *C. parapsilosis* complex, respectively. *Candida orthopsilosis* is a rare *Candida* species that reported by Tavanti et al. [33] for the first time. We also identified this species for the first time in Iran in our previous study [34]. In agreement with our previous report, here it was isolated from a patient with fingernail infection. *Trichosporon asahii (Trichosporon beigelii)* was another uncommon yeast isolated in the present study and identified by PCR-sequencing. This species is connected with a wide spectrum of clinical signs, ranging from superficial lesions in immunocompetent individuals to severe systemic and fatal infections in immunocompromised patients [35]. It is the most prevalent non-*Candida* cause of fungemia [36], however, all *T. asahii* in the present study were isolated from urine samples. We also isolated *Pichia terricola* from clinical sample for the first time. *Pichia* genus is found in plants, fruit juices, and soil. It has also been described as a normal flora of throat, skin, and alimentary tract. Some species such as *P. anomala* can cause serious infections among immunosuppressed patients mainly in infants [37], however, we isolated *P. terricola* from the urine sample of a pregnant female with severe urinary tract infection.

In conclusion, the contribution of *C. albicans* and NAC species in *Candida* infections is changing from the past in different areas, and epidemiological data show the increasing of NAC infections worldwide. Due to the increasing of antifungal resistance of *Candida* species and the elevating number of immuno suppressed patients, it is essential to provide new effective strategies for treatment of this fungal infection. As NAC species have various virulence factors and antifungal susceptibility profile, precise molecular identification can help us to reach to these advantageous strategies.

## Limitations

Due to the sanctions, the most antifungal agents were not found in the country, and if they were, they would be 4–5 times of the price. We recommend the antifungal susceptibility testing of clinical isolates in further studies.

## Supplementary information

**Additional file 1.** Agarose gel electrophoresis photos of PCR–RFLP taken from primary screening and repeat experiments. **Fig S1.** Agarose gel electrophoresis for PCR–RFLP; from left to right: *C. albicans, C. glabrata, C. albicans, C. glabrata, C. glabrata, C. albicans, C. glabrata, C. albicans, C. glabrata*, and 100 bp DNA size Marker. **Fig S2.** Agarose gel electrophoresis for PCR–RFLP; from left to right: *C. glabrata, C. parapsilosis, C. tropicalis, C. albicans, C. albicans*, No band, *C. albicans, C. albicans* (dim band), and 100 bp DNA size Marker. **Fig S3.** Agarose gel electrophoresis for PCR–RFLP; from left to right: *C. albicans, C. tropicalis, C. albicans, C. albicans, C. albicans*, Mixed bands (*C. albicans, and C. kefyr*), *C. albicans, C. albicans, C. albicans, C. albicans, C. albicans, C. glabrata, C. glabrata, C. tropicalis, C. albicans*, and 100 bp DNA size Marker. **Fig S4.** Agarose gel electrophoresis for PCR–RFLP; from left to right: *C. parapsilosis, C. parapsilosis, C. parapsilosis, C. parapsilosis, C. parapsilosis,* No band, and 100 bp DNA size Marker. **Fig S5.** Agarose gel electrophoresis for PCR–RFLP; from left to right: *C. albicans, C. albicans, C. albicans, C. albicans, C. albicans, C. albicans, C. albicans, C. albicans, C. albicans*, and 100 bp DNA size Marker. **Fig S6.** Agarose gel electrophoresis for PCR–RFLP; from left to right: *C. tropicalis*, *C. parapsilosis, C. albicans, albicans, albicans*, and 100 bp DNA size Marker.

## Data Availability

All data generated or analyzed during this study are included in this article.

## Abbreviations

NAC: non-*albicans Candida*
MIC: minimum inhibitory concentration
PCR: polymerase chain reaction
RFLP: restriction fragment length polymorphism
