# Supplementary material for: An alarming rise of non-albicans Candida species and uncommon yeasts in the clinical samples; a combination of various molecular techniques for identification of etiologic agents
Source: BMC Res Notes. 2019 Nov 29;12:779. doi: 10.1186/s13104-019-4811-1 (PMC6883655; doi:10.1186/s13104-019-4811-1)
Supplement: Supplementary file 1 — Additional file 1. Agarose gel electrophoresis photos of PCR–RFLP taken from primary screening and repeat experiments. Fig S1. Agarose gel electrophoresis for PCR–RFLP; from left to right: C. albicans, C. glabrata, C. albicans, C. glabrata, C. glabrata, C. albicans, C. glabrata, C. albicans, C. glabrata, and 100 bp DNA size Marker. Fig S2. Agarose gel electrophoresis for PCR–RFLP; from left to right: C. glabrata, C. parapsilosis, C. tropicalis, C. albicans, C. albicans, No band, C. albicans, C. albicans (dim band), and 100 bp DNA size Marker. Fig S3. Agarose gel electrophoresis for PCR–RFLP; from left to right: C. albicans, C. tropicalis, C. albicans, C. albicans, C. albicans, Mixed bands (C. albicans, and C. kefyr), C. albicans, C. albicans, C. albicans, C. albicans, C. albicans, C. glabrata, C. glabrata, C. tropicalis, C. albicans, and 100 bp DNA size Marker. Fig S4. Agarose gel electrophoresis for PCR–RFLP; from left to right: C. parapsilosis, C. parapsilosis, C. parapsilosis, C. parapsilosis, C. parapsilosis, No band, and 100 bp DNA size Marker. Fig S5. Agarose gel electrophoresis for PCR–RFLP; from left to right: C. albicans, C. albicans, C. albicans, C. albicans, C. albicans, C. albicans, C. albicans, C. albicans, C. albicans, and 100 bp DNA size Marker. Fig S6. Agarose gel electrophoresis for PCR–RFLP; from left to right: C. tropicalis, C. parapsilosis, C. albicans, albicans, albicans, and 100 bp DNA size Marker. [file 13104_2019_4811_MOESM1_ESM.docx]

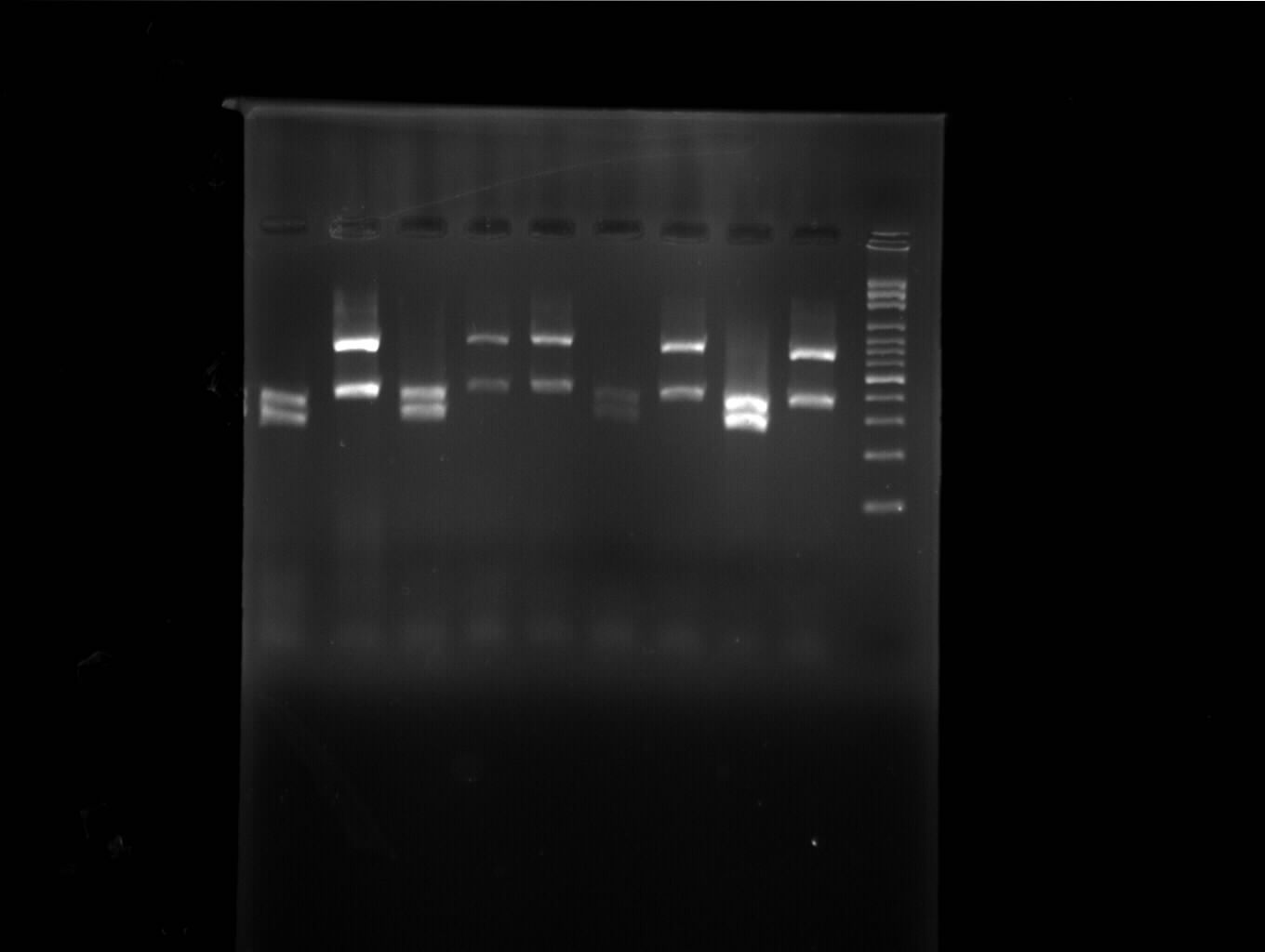


Fig S1. Agarose gel electrophoresis for PCR-RFLP; from left to right: *C. albicans, C. glabrata, C. albicans, C. glabrata, C. glabrata, C. albicans, C. glabrata, C. albicans, C. glabrata*, and 100 bp DNA size Marker


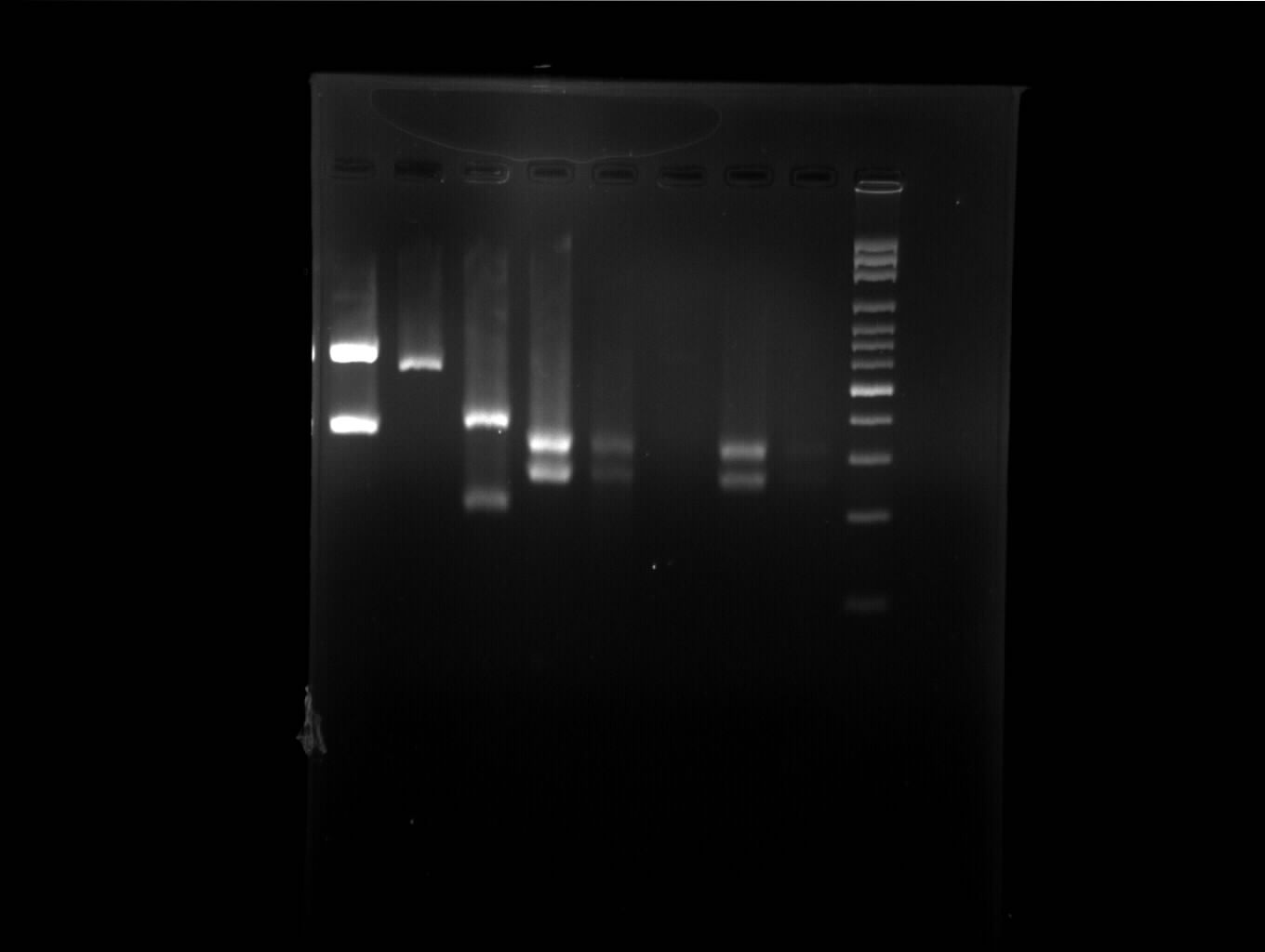


Fig S2. Agarose gel electrophoresis for PCR-RFLP; from left to right: *C. glabrata, C. parapsilosis, C. tropicalis, C. albicans, C. albicans*, No band, *C. albicans, C. albicans* (dim band), and 100 bp DNA size Marker


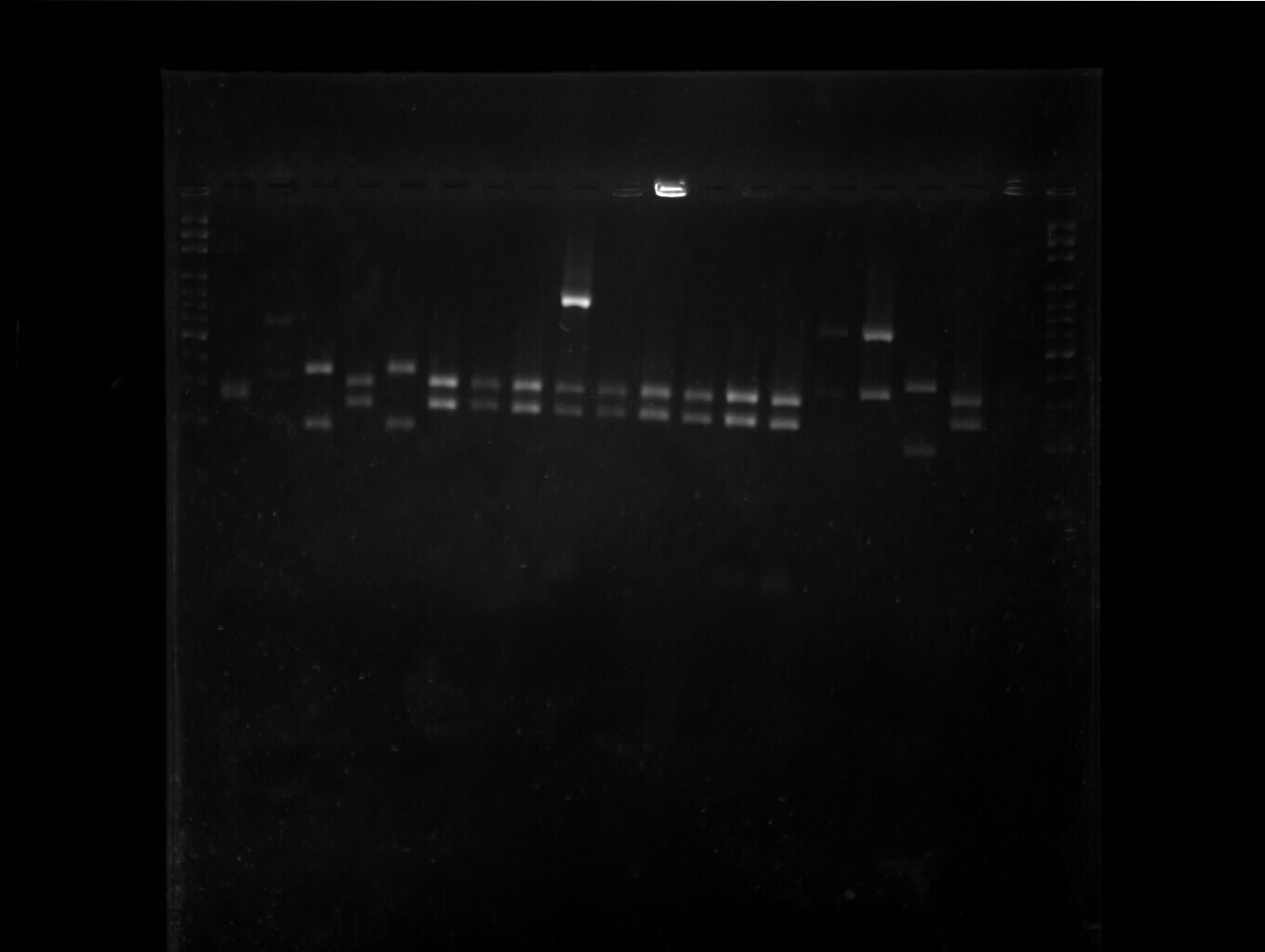


Fig S3. Agarose gel electrophoresis for PCR-RFLP; from left to right: *C. albicans, C. tropicalis, C. albicans, C. albicans, C. albicans*, Mixed bands (*C. albicans, and C. kefyr*), *C. albicans, C. albicans, C. albicans, C. albicans, C. albicans, C. glabrata, C. glabrata, C. tropicalis, C. albicans*, and 100 bp DNA size Marker

Repeat experiments

In this step, we selected 40 strains including *Candida albicans, C. tropicalis, C. kefyr, C. parapsilosis* complex, and *C. glabrata* complex randomly, and repeated PCR-RFLP test to confirm our primary findings. All results in this stage were consistent with the findings in the first stage.


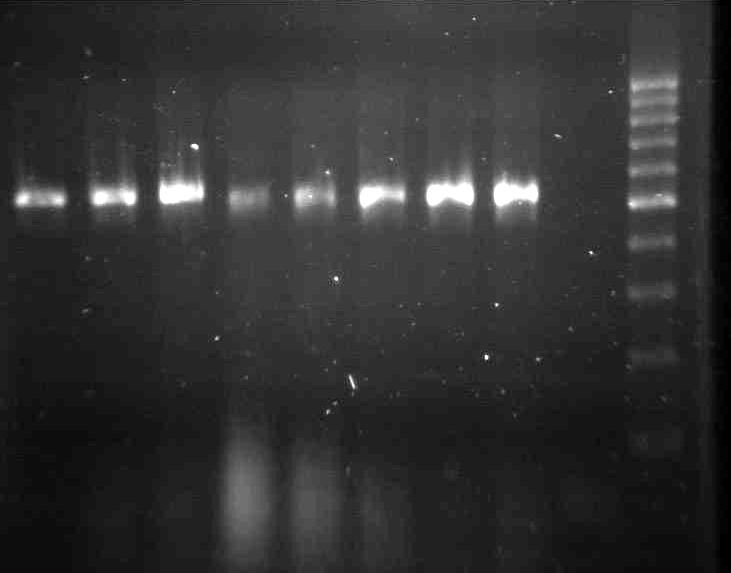


Fig S4. Agarose gel electrophoresis for PCR-RFLP; from left to right: *C. parapsilosis, C. parapsilosis, C. parapsilosis, C. parapsilosis, C. parapsilosis,* No band, and 100 bp DNA size Marker

Repeat experiments


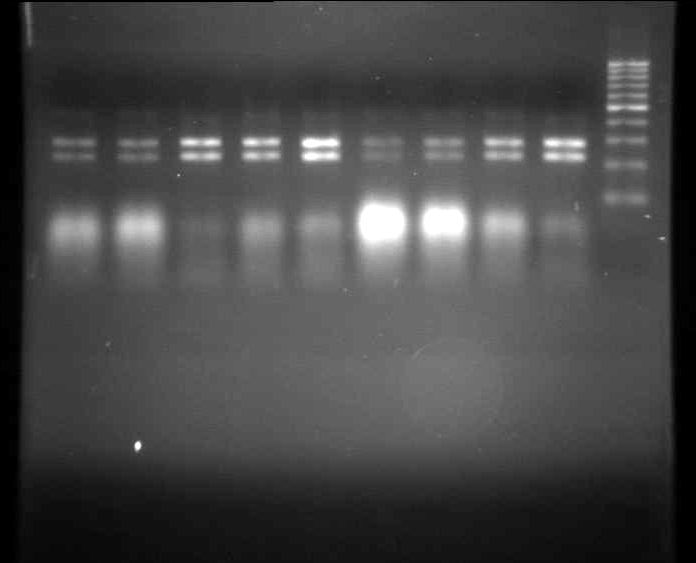


Fig S5. Agarose gel electrophoresis for PCR-RFLP; from left to right: *C. albicans, C. albicans, C. albicans, C. albicans, C. albicans, C. albicans, C. albicans, C. albicans, C. albicans*, and 100 bp DNA size Marker

Repeat experiments


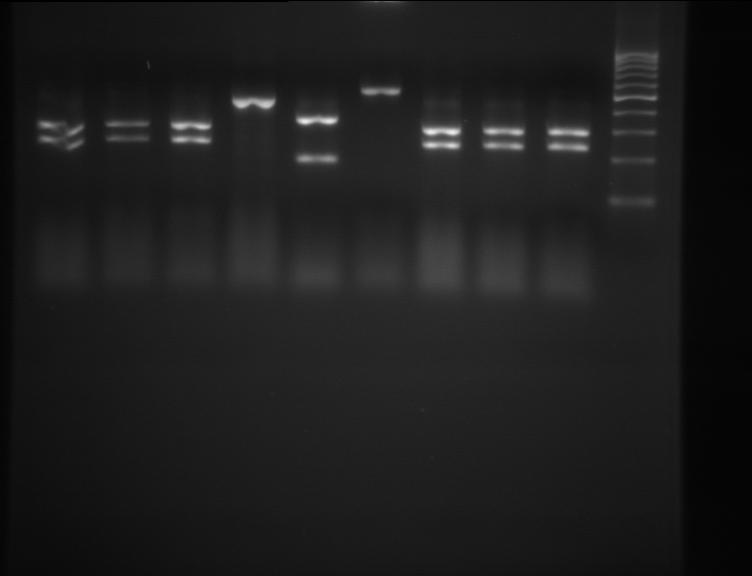


Fig S6. Agarose gel electrophoresis for PCR-RFLP; from left to right: *C. tropicalis, C. parapsilosis, C. albicans, albicans, albicans,*, and 100 bp DNA size Marker
